# Comprehensive analysis of a decade of cumulative radiocesium testing data for foodstuffs throughout Japan after the 2011 Fukushima Daiichi Nuclear Power Plant accident

**DOI:** 10.1371/journal.pone.0274070

**Published:** 2022-09-21

**Authors:** Kosuke Nakamura, Shinji Chiba, Takashi Kiuchi, Hiromi Nabeshi, Tomoaki Tsutsumi, Hiroshi Akiyama, Akiko Hachisuka

**Affiliations:** National Institute of Health Sciences, Kawasaki, Kanagawa, Japan; Northwestern University Feinberg School of Medicine, UNITED STATES

## Abstract

The unexpected accident at the Fukushima Daiichi Nuclear Power Station in Japan, which occurred on March 11th, 2011, after the Great East Japan Earthquake and tsunami struck the north-eastern coast of Japan, released radionuclides into the environment. Today, because of the amounts of radionuclides released and their relatively long half-life, the levels of radiocesium contaminating foodstuffs remain a significant food safety concern. Foodstuffs in Japan have been sampled and monitored for ^134,137^Cs since the accident. More than 2.5 million samples of foodstuffs have been examined with the results reported monthly during each Japanese fiscal year (FY, from April 1^st^ to March 31^st^) from 2012 to 2021. A total of 5,695 samples of foodstuffs within the “general foodstuffs” category collected during this whole period and 13 foodstuffs within the “drinking water including soft drinks containing tea as a raw material” category sampled in FY 2012 were found to exceed the Japanese maximum permitted level (JML) set at 100 and 10 Bq/kg, respectively. No samples from the “milk and infant foodstuffs” category exceeded the JML (50 Bq/kg). The annual proportions of foodstuffs exceeding the JML in the “general foodstuffs” category varied between 0.37% and 2.57%, and were highest in FY 2012. The ^134,137^Cs concentration for more than 99% of the foodstuffs monitored and reported has been low and not exceeding the JML in recent years, except for those foodstuffs that are difficult to cultivate, feed or manage, such as wild mushrooms, plants, animals and fish. The monitoring data for foodstuffs show the current status of food safety risks from ^134,137^Cs contamination, particularly for cultured and aquaculture foodstuffs on the market in Japan.

## Introduction

Radionuclides were released to the environment following an accident on March 11^th^, 2011, at the Fukushima Daiichi Nuclear Power Station (FDNPS) run by the Tokyo Electronic Power Company in Japan, which occurred after the Great East Japan Earthquake and tsunami struck the north-eastern coast of Japan. The reported levels of radionuclides released differed according to their volatility **[1]**, with volatile noble gases, such as xenon and krypton, and others such as iodine (I), cesium (Cs) and tellurium being released **[2]**. However, the release of low volatile elements, such as strontium, barium, uranium and plutonium, was much lower **[1, 3]**. In 2020, the United Nations Scientific Committee on the Effects of Atomic Radiation (UNSCEAR) concluded that ^131^I (half-life [*t*_1/2_] 8.02 d), ^134^Cs (*t*_1/2_ 2.06 y) and ^137^Cs (*t*_1/2_ 30.2 y) were the greatest contributors to released radionuclides, with the total release of relatively long-life ^131^I and ^137^Cs being reported as 100-500 and 6-20 PBq, about 10% and 20% of the levels estimated from the Chernobyl Nuclear Power Station accident in 1986, respectively **[1]**.

Considering the amount released and the *t*_1/2_ of the radionuclides, ^134^Cs and ^137^Cs (^134,137^Cs) are the major concern for the contamination of foodstuffs more than a decade after the FDNPS accident **[1]**. Most of the released ^134,137^Cs is dispersed but a substantial proportion persists in a resuspension-deposition cycle within the land mass. Consequently, the monitoring of ^134,137^Cs in organisms may need to continue until they decay naturally or dissipate in the environment to undetectable levels **[4–11]**. The processes of the resuspension of ^134,137^Cs are not fully understood but many studies have concluded that the concentrations and exposure levels of radioactive Cs were well below regulatory levels, and that their contribution to the long-term exposure of the public from consuming foodstuffs has not been significant in recent years **[12–14]**.

The extent of ^134,137^Cs contamination for foodstuffs in Japan after the FDNPS accident has been examined according to monitoring guidelines established by the nuclear emergency response headquarters of Japan **[15]**. The ^134,137^Cs concentrations were measured in foodstuffs and the data reported by local governments in Japan according to the food hygiene monitoring guidance plan following the Food Sanitation Act **[16]**. The monthly raw data were collected, summarized then released to the public by the Ministry of Health, Labour and Welfare of Japan (MHLW) **[17]**. The nuclear emergency response headquarters in Japan has reviewed the food hygiene monitoring guidance plan for testing foodstuffs **[18]**, and updates have been made almost annually since 2011 so that the rationality and efficiency of the inspections have been steadily improved. Acting on changes in the monitoring results has allowed scientific knowledge to accumulate and improve, enabling, for example, the status of cases of cancellation of shipping restrictions to be reassessed **[19, 20]**. Many countries or regions have now lifted their restrictions on importing Japanese foodstuffs but some still continue to impose regulations. Currently, analysis of the risk from consuming foodstuffs contaminated with radioactive substances has not completely addressed consumers’ concerns, both domestically and internationally. Consumers remain concerned about residual radioactive contamination especially in seafood **[21, 22]**. Future monitoring plans need to take into account these concerns as well as the accumulated scientific knowledge gained by monitoring levels of contamination in the years since the FDNPS accident. Critical scientific advice is still needed on which types of foodstuff should be the focus of examination, so that the successful risk analysis of such unexpected accidents can be prioritized.

In the present study, we analyzed more than a decade of accumulated ^134,137^Cs monitoring data on foodstuffs that have been sampled throughout Japan. From these data, the chronological changes in ^134,137^Cs concentration data in a range of Japanese foodstuffs have been investigated using comprehensive data analyses. These ^134,137^Cs nationwide monitoring data will show the current status of food safety regarding contamination by radionuclides, particularly for cultured and aquaculture foodstuffs that are available in Japan.

## Materials and methods

### Accessing data sources

The ^134,137^Cs monitoring data were reported by the prefectural governments around Japan, with the publicly available monthly reports obtained as Microsoft Excel files from the MHLW website **[23]**. For screening foodstuffs for ^134,137^Cs concentrations at < 50 Bq/kg, the monitoring data were obtained according to the Japanese official testing methods using gamma spectrometry with sodium iodide, NaI(Tl), and cesium iodide CsI(Tl) scintillation detectors **[24, 25]**. For detecting ^134,137^Cs concentrations at > 50 Bq/kg in foodstuffs, a Ge semiconductor detector was used. For dried processed foodstuffs, such as dried mushrooms, the moisture content data (percentage weight change) were taken into account; otherwise, they were examined directly in the dried state **[26]**. The data obtained from the database were originally in Japanese so were converted to alphabetical text and numeric data for direct computation using the Python programming language. All data reported from April 2012 to March 2022 were acquired from the website using Anaconda 3 (Ver. 2020.02) with Python 3 (Ver. 3.7.6 for Ubuntu) software. The web page scraping tool, Beautiful Soup 4 (Ver. 4.8.2), and the HTTP operation tool, urllib3 (Ver. 1.25.8), were used for the website structural analysis and extracting the URL-linked elements, with these software tools being operated using Jupyter Notebook.

### Data analysis

The monthly data files were concatenated and integrated into annually summarized data files using Python 3 and the pandas (Ver. 1.2.3) package. To compute the text format and numeric data, the “drop” and “dropna” methods were used to remove unnecessary blank lines in the data. The “loc” and “iloc” attributes were then used to extract the required lines and columns during the concatenation and integration. The “replace” method was used when deleting unnecessary characters in a numeric column or replacing obvious text errors. In addition, the “regex” argument was used when replacing a character string. A specific character string was corrected by combining pattern matching of the character string by regular expression with an exact and partial match. Methods such as “where” and “merge” were used in combination when each sample was categorized into a specific food group. The “pandas.to_datetime” method was used to convert the reported date data for analysis. The obtained data were basically classified by the “Sampling Date” reported in the original data as the reported concentration data were decay-corrected to the sampling date. Samples with no “Sampling Date” information were classified by “Results Obtained Date”, and samples with no “Sampling Date” and no “Results Obtained Date” were classified by the “Press Release Date”. All dates indicated in “Sampling Date”, “Results Obtained Date” and “Press Release Date” were corrected and summarized from that originally reported in the FY as a Japanese era name to the period Anno Domini (AD). Numeric items recognized as character strings were converted to numeric data using the “pandas.to_numeric” method. After the correction and pre-processing, the data were saved in a comma-separated value (CSV) file by the “pandas.to_csv” method. The number of samples in the data was assessed by counting lines.

In the present study, each ^134,137^Cs concentration reported for a foodstuff was compared with the Japanese maximum permitted level (JML) values set for three food categories: 10 Bq/kg for “drinking water including soft drinks containing tea as a raw material (For the liquid tea, samples were reported to be prepared by adding 30 times volume of 90°C water to 10 g of dried tea leaves and filtering through 40-mesh filter after extracting for 60 seconds); 50 Bq/kg for the “milk and infant foodstuffs”; and 100 Bq/kg for “general foodstuffs” that includes livestock, agricultural and fishery foodstuffs, wild birds and animals and others such as processed food commodities **[15]**. For “general foodstuffs”, samples in the original monthly report at more than or equal to 110 Bq/kg were reported to exceed the JML value. The threshold value for detecting ^134,137^Cs concentrations in the “general foodstuffs” category was set at a quarter of the JML value, i.e., samples at 25 Bq/kg or less were considered negative (“not detected”). For fishery foodstuffs, data on freshwater fishery foodstuffs were extracted from the data, and others, including marine fishery foodstuffs, crustaceans, shellfish, seaweeds and migratory fish, were then extracted as “not freshwater fishery foodstuffs” (**S1 Fig**). Samples with no information on their origin (wild or aquaculture) were included into the wild origin class in the data analyses.

For data aggregation, R (Ver. 4.0.3) and RStudio Server (Ver. 1.3.1093) with tidyverse (Ver. 1.3.0), dplyr (Ver. 1.0.5), tidyr (Ver. 1.1.3), ggplot2 (Ver. 3.3.3), lubridate (Ver. 1.7.10), stringr (Ver. 1.4.0), knitr (Ver. 1.31), readr (Ver. 1.4.0) and extrafont (Ver. 0.17) packages were used. The obtained CSV files were read using the readr package and concatenated using the rbind function. Other categorical variable items, such as “prefecture”, “marketed,” “non-marketed” (ones that include those produced for sale or not for sale, but that have not been in market), “food category”, “food category 2”, “food classification”, “inspection instrument”, “region classification” and “exceed action levels”, were factorized into categories using the “as.factor” function. For data extraction, the “subset”, “filter” and “group_by” functions, in combination with general R functions, such as “summarise”, “spread”, “count” and “xtabs”, were used as well as Microsoft Excel’s pivot tables.

### Visualization of results

The data were analyzed using RStudio server (Ver. 1.3.1093). Additional packages including ggplot2 (Ver. 3.3.3) for graph drawing, knitr (Ver. 1.31) for table processing and the Japanese TrueType font compatible package extrafont (Ver. 0.17) were used. Scatter plots were obtained using the “geom_point” function in the ggplot2 package. Histograms were created using the “geom_histgram” function. For the distribution of the detected ^134,137^Cs concentration values, a violin plot was created using the “geom_violin” function. Mapping data were made with Natural Earth. Briefly, to locate the reported samples in the dataset, the information on “prefecture” was used to match with the addresses of the local governments’ head offices then the locations were plotted on maps (shape files) downloaded from Natural Earth (https://www.naturalearthdata.com/, last accessed on January 21, 2022) using the “geom_sf” function.

### Statistical methods

Fisher’s exact test was applied to compare the annual sample numbers. Bonferroni adjustment was applied for multiple comparisons. Calculated corrected p-values were considered statistically significant at < 0.05 in this study. The statistical analyses were performed using R.

## Results and discussion

### Data characterization

To analyze any chronological trends observed from the ^134,137^Cs concentration data for the examined foodstuffs after the accident, the data obtained from the MHLW webpage were first compiled into one set of annual data. Because the food hygiene monitoring guidance plan for testing foodstuffs in Japan is updated annually according to the Japanese fiscal year (April 1^st^ to March 31^st^ of the next year), the compiled data following the FY were analyzed comprehensively according to the scheme in **S2 Fig**. **Table 1** summarizes the data according to the detailed attributes reported for each sample. The samples in the dataset were separately analyzed in three reported categories, “drinking water including soft drinks containing tea as a raw material”, “milk and infant foodstuffs” and “general foodstuffs”, which were set at different JML values of 10, 50 and 100 Bq/kg, respectively, based on the level for an individual’s annual internal dose through food consumption of 1 mSv, as recommended by Codex Alimentarius **[27]**. The data included the reports on the 2,578,532 samples examined, including 6,314 from the “drinking water including soft drinks containing tea as a raw material”, 30,050 from the “milk and infant foodstuffs” and 2,542,168 from the “general foodstuffs” categories. The total number of samples reported from all 47 prefectures in Japan from the FY 2012 to 2019 ranged from 283,194 to 343,504 per year. During the FY 2012, when the screening method for ^134,137^Cs in foodstuffs was officially launched in March 2012 with the implementation of the current JML values from April 2012, data from 283,194 samples were reported. During the FY 2019, 288,725 samples were reported then after testing meat from all livestock ended, the number of samples reported since the FY 2020 declined dramatically in many prefectures. The breakdown of the data in terms of the reported information on market distribution conditions resulted in 175,612 marketed and 2,402,915 non-marketed foodstuffs that were produced for sale and not for sale, respectively, including the “drinking water and soft drinks containing tea as a raw material” category. Overall, the dataset from the decade included data from more than 2.5 million samples that were examined and reported throughout Japan according to the official testing methods of Japan **[24, 25]** (**S1–S4 Tables, S3–S6 Figs**).

**Table 1:** Summary of ^134,137^Cs monitoring data from April 2012 to March 2021*.

Fiscal year	No. of samples reported	Sale condition (rate, %)**										Food categories (rate, %) **														Cs concentration reported (rate, %) **				No. of foods in the Cs concentration range (rate, %) **							
		Marketed products		Non-marketed products						Others***		General foods										Milk, Infant foods		Drinking water		with defined number		with inequality sign		>100 Bq/kg		>50 Bq/kg		>25 Bq/kg		≤25 Bq/kg	
						Produce for sale		Produce not for sale				Fishery products		Livestock products		Agricultural products		Wild animal meat		Others (including processed foods)																	
2012	283,194	26,653	(1.0)	256,541	(10.0)	NA	NA	NA	NA	NA	NA	21,397	(0.83)	192,929	(7.48)	50,380	(1.95)	1,376	(0.05)	10,166	(0.39)	5,258	(0.20)	1,688	(0.07)	24,229	(0.94)	258,965	(10.04)	2,428	(0.09)	2,984	(0.12)	27,108	(1.05)	250,674	(9.72)
2013	337,754	29,709	(1.2)	308,045	(12.0)	NA	NA	NA	NA	NA	NA	23,054	(0.89)	249,133	(9.66)	46,705	(1.81)	1,364	(0.05)	11,265	(0.44)	5,093	(0.20)	1,140	(0.04)	18,643	(0.72)	319,111	(12.38)	997	(0.04)	2,024	(0.08)	2,513	(0.10)	332,220	(12.88)
2014	314,887	26,215	(1.0)	288,669	(11.2)	NA	NA	NA	NA	3	(0.0)	23,583	(0.91)	237,328	(9.20)	36,642	(1.42)	1,415	(0.05)	10,600	(0.41)	4,512	(0.17)	807	(0.03)	11,459	(0.44)	303,428	(11.77)	589	(0.02)	790	(0.03)	1,563	(0.06)	311,945	(12.10)
2015	343,504	22,115	(0.9)	321,388	(12.5)	NA	NA	NA	NA	1	(0.0)	20,614	(0.80)	278,708	(10.81)	29,127	(1.13)	1,020	(0.04)	9,803	(0.38)	3,661	(0.14)	571	(0.02)	7,896	(0.31)	335,608	(13.02)	424	(0.02)	461	(0.02)	1,064	(0.04)	341,555	(13.25)
2016	323,313	18,932	(0.7)	304,380	(11.8)	NA	NA	NA	NA	1	(0.0)	19,834	(0.77)	263,775	(10.23)	25,810	(1.00)	1,519	(0.06)	8,643	(0.34)	3,219	(0.12)	513	(0.02)	7,415	(0.29)	315,898	(12.25)	301	(0.01)	442	(0.02)	1,197	(0.05)	321,373	(12.46)
2017	308,277	15,409	(0.6)	292,868	(11.4)	NA	NA	NA	NA	NA	NA	18,042	(0.70)	258,537	(10.03)	20,344	(0.79)	1,684	(0.07)	6,835	(0.27)	2,407	(0.09)	428	(0.02)	5,904	(0.23)	302,373	(11.73)	211	(0.01)	310	(0.01)	853	(0.03)	306,903	(11.90)
2018	302,038	13,295	(0.5)	288,683	(11.2)	60	(0.0)	NA	NA	NA	NA	14,768	(0.57)	259,290	(10.06)	17,394	(0.67)	2,240	(0.09)	5,814	(0.23)	2,121	(0.08)	411	(0.02)	5,650	(0.22)	296,388	(11.49)	304	(0.01)	414	(0.02)	957	(0.04)	300,363	(11.65)
2019	288,725	10,656	(0.4)	260,098	(10.1)	17,877	(0.7)	94	(0.0)	NA	NA	13,161	(0.51)	251,879	(9.77)	14,392	(0.56)	2,681	(0.10)	4,407	(0.17)	1,842	(0.07)	363	(0.01)	5,059	(0.20)	283,666	(11.00)	167	(0.01)	205	(0.01)	808	(0.03)	287,545	(11.15)
2020	40,765	7,060	(0.3)	340	(0.0)	30,236	(1.2)	3,129	(0.1)	NA	NA	10,793	(0.42)	8,868	(0.34)	13,494	(0.52)	3,420	(0.13)	2,939	(0.11)	1059	(0.04)	192	(0.01)	4,535	(0.18)	36,230	(1.41)	121	(0.00)	187	(0.01)	741	(0.03)	39,716	(1.54)
2021	36,075	5,568	(0.2)	2	(0.0)	27,577	(1.1)	2,928	(0.1)	NA	NA	11,941	(0.46)	7,178	(0.28)	11,751	(0.46)	1,852	(0.07)	2,274	(0.09)	878	(0.03)	201	(0.01)	3,567	(0.14)	32,508	(1.26)	153	(0.01)	187	(0.01)	519	(0.02)	35,216	(1.37)
Subtotal	2,578,532	175,612		2,321,014		75,750		6,151		5		177,187		2,007,625		266,039		18,571		72,746		30,050		6,314		94,357		2,484,175		5,695		8,004		37,323		2,527,510	
				2,402,915								2,542,168																		2,578,532							
Total 2,578,532																																					

*NA, no data available. The data collection method changed in May, 2018. All data using all instruments used including no indicated instrument and a non-destructive method reported in the database were summarized.

**The rate was calculated using the total no. of samples reported.

***Samples without information.

For an overview of the annual trends of ^134,137^Cs in all reported foodstuffs, the reported ^134,137^Cs concentration values were examined (**Table 1**, **S7A Fig**). Within the period examined, 37,323 cases (1.45%) were reported as exceeding 25 Bq/kg, which was set as the threshold of radioactive Cs detection in the present study. The samples in the dataset were then separately analyzed in three categories, “drinking water including soft drinks containing tea as a raw material”, “milk and infant foodstuffs” and “general foodstuffs”. **S5 Table** shows the number of samples, in which ^134,137^Cs was detected and the reported concentration exceeding the JML value for each foodstuff category. By using a germanium (Ge) semiconductor detector to aggregate only those sample results whose concentrations were confirmed by a more precise analysis (**S8 Fig**), the 5,688 cases exceeding the JML values from the FY 2012 to FY 2021 were placed in the “general foodstuff” category. From the same category, 7 remaining cases sampled in the FY 2018 were detected as exceeding JML values using the NaI(Tl) scintillation detector. Thus, a total of 5,695 cases (0.22% of all examined cases) was reported as exceeding 100 Bq/kg. Thirteen samples of prepared liquid tea with more than 10 Bq/kg (without inequality sign) in the “drinking water including soft drinks containing tea as a raw material” category were found to exceed the JML value by the Ge semiconductor detector in a single year, FY 2012. None of the “milk and infant foodstuffs” samples exceeding the JML value were reported at more than 50 Bq/kg. Of all the samples, the highest value for samples exceeding the JML was 61,000 Bq/kg in non-marketed wild boar meat in the “general foodstuffs” category reported from Minamisoma city in Fukushima prefecture (Food classification no. 21878, sampled on March 11, 2013). The lowest value was 11 Bq/kg in two drinking teas in the “drinking water including soft drinks containing tea as a raw material” category reported from Rikuzentakata city in Iwate prefecture (Food classification no. 5403, sampled on May 29, 2012) and from Omitama city in Ibaraki prefecture (Food classification no. 12182, sampled on May 16, 2012) (**S7B Fig**). **Table 1** and **S5 Table** show that more than 98.0% and 99.8%, respectively, of all of the samples tested were below the detection limit (25 Bq/kg) and did not exceed the JML. Subsequently, there was no apparent significant change in the distribution of such low reported ^134,137^Cs concentrations in each FY (**S7B Fig**).

### Reported foodstuffs exceeding the JML

**Fig 1:**
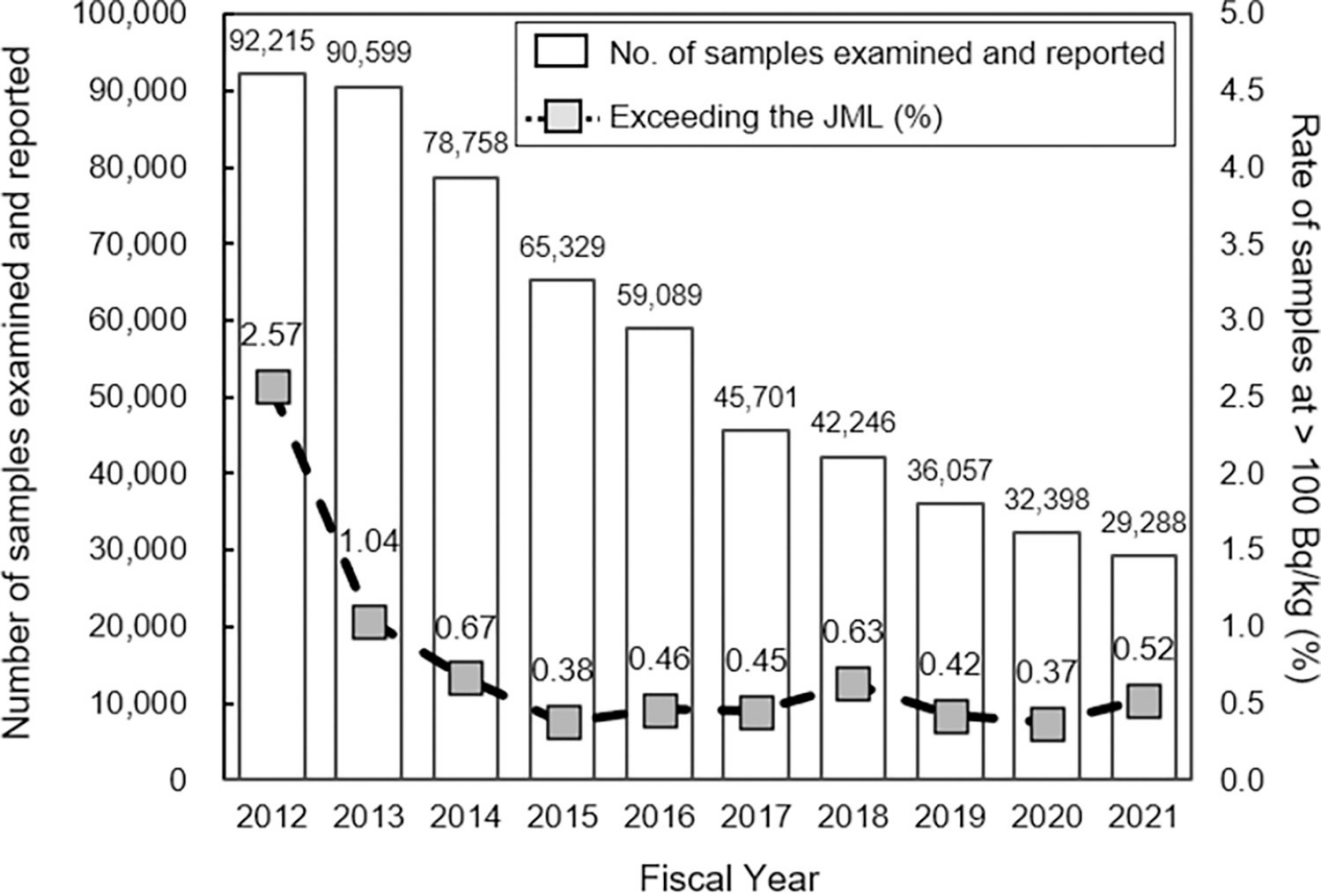
The “general foodstuffs” examined and reported exceeding the JML of 100 Bq/kg from FY 2012 to 2021. The number of reported samples excluding meat from all livestock is indicated above the bar. The proportion of samples exceeding 100 Bq/kg within each year were calculated.

To explore the reported information in the “general foodstuffs” category that exceeded the JML, samples with ^134,137^Cs concentrations above 100 Bq/kg were extracted from the compiled data. In the “general foodstuffs” category, between 7.48% and 10.81% of these samples tested were cattle meats from all livestock testing practice until the FY 2019. **Fig 1** shows the number of reported samples exceeding 100 Bq/kg over time specifically excluding cattle meats from all livestock testing practice. The number of samples with ^134,137^Cs concentrations exceeding 100 Bq/kg declined each year from the FY 2012 to FY 2015. The relative frequency rate from the annual data decreased each year and varied significantly between the FY 2012 and FY 2015 (assuming that data were collected and tested for ^134,137^Cs concentrations annually under the same conditions throughout Japan; Fisher’s exact test, p < 0.05). The detailed sample information exceeding JML from the “general foodstuffs” category was extracted from the data and is summarized in **S6 Table**. Most of the foodstuffs (excluding meat from all livestock) were non-marketed (**S9 Fig**). **S9 Fig** shows that the number of non-marketed foodstuffs exceeding the JML decreased each year and varied significantly between the FY 2012 and FY 2015 (assuming that the data were collected and tested for ^134,137^Cs concentrations annually under the same conditions throughout Japan; Fisher’s exact test, p < 0.05). In contrast, the proportion exceeding the JML in marketed foodstuffs tended to fluctuate slightly and did not vary significantly between the FY 2013 and FY 2015 (assuming the data were collected and tested for ^134,137^Cs concentrations annually under the same conditions throughout Japan; Fisher’s exact test, p > 0.05). This trend seems reasonable because foodstuffs collected as “non-marketed” included those collected not only before pre-marketing, but also those for cases of inspection before reconsideration of lifting shipping restrictions or testing operations. In the FY 2020 to FY 2021, the proportion exceeding the JML increased in the marketed foodstuffs, because the number of tests increased on wild animals, plants (such as Koshiabura), mushrooms and honey, that were frequently found to exceed the JML. This may an indicative of a change in monitoring strategy in Japan, with the focus on their monitoring according to the food hygiene monitoring guidance plan for testing foodstuffs **[18]**.

**Fig 2:**
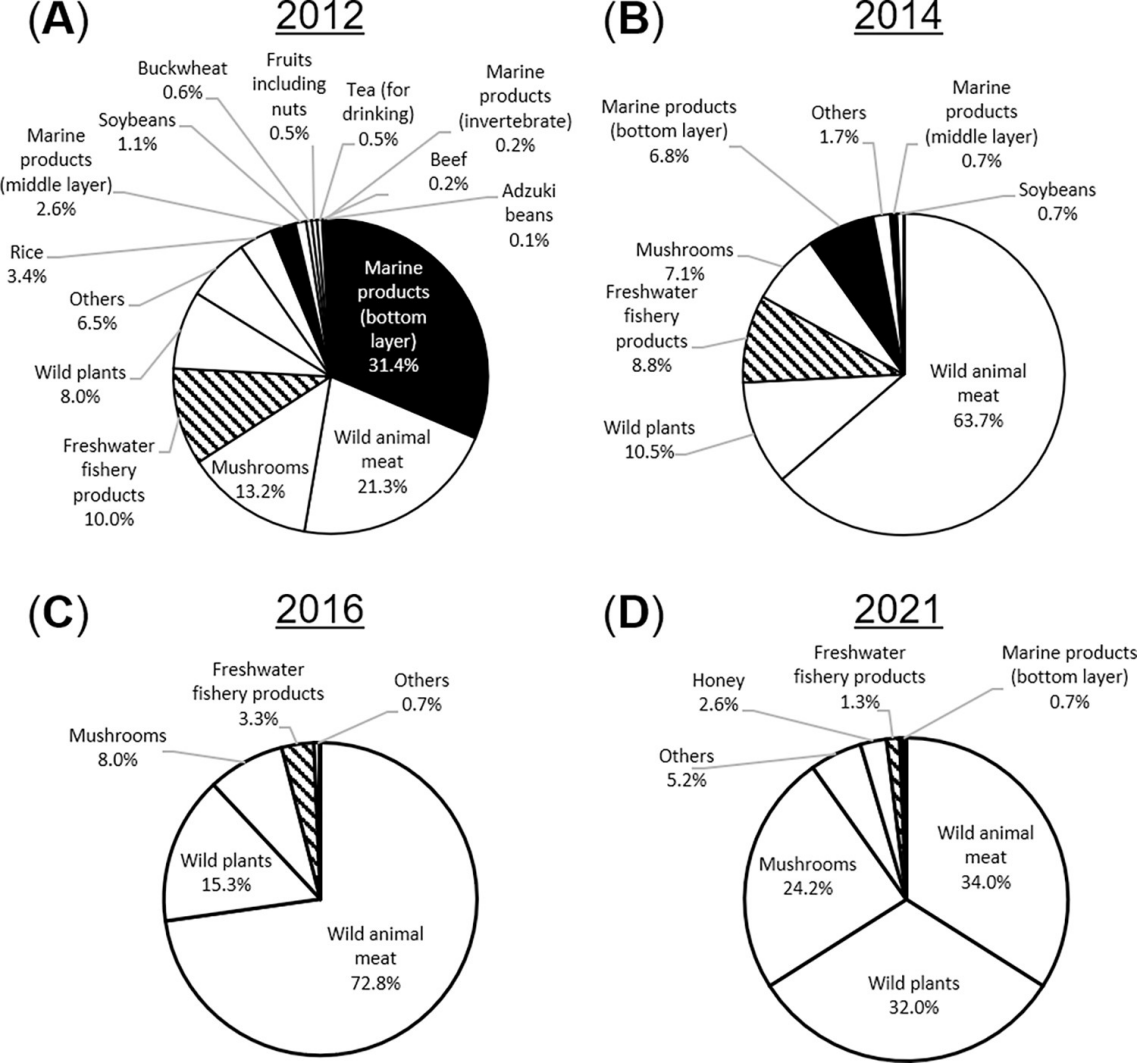
Annual trends of the “general foodstuffs” reported exceeding the JML (> 100 Bq/kg). Food categories examined and reported as exceeding 100 Bq/kg. Pie graphs show the proportions of food categories reported in each FY: (**A**) 2012; (**B**) 2014; (**C**) 2016; and (**D**) 2021. Freshwater fishery foodstuffs and other fishery foodstuffs including marine products are shaded with lines and filled in black, respectively. All data reported in the database were included in the analysis.

**Fig 2** summarizes the data obtained from the MHLW webpage for the foodstuffs examined and reported with ^134,137^Cs concentrations exceeding 100 Bq/kg, with the types of foodstuff reported being observed to change over time. Specifically, most of the samples exceeding the JML were reported to be marine fishery foodstuffs in the FY 2012. However, in the FY 2013, the type changed to wild harvested foods, such as wild animal meat, plants and mushrooms. The samples in the FY 2012–2021 were further categorized and summarized in **S10 Fig**. Since FY 2015, the samples exceeding the JML have been continuously reported (**Fig 1**). The main foodstuffs exceeding the JML were wild harvested foods and dried processed food products, such as dried shiitake mushrooms, anpo-gaki (semi-dried persimmons), pickled plums and mulberry tea, where the ^134,137^Cs had been concentrated because of the reduction in sample mass during food processing. Most of the foodstuffs were wild harvested that had not been bred or managed (fishery foodstuffs such as marine bottom layer and freshwater fish, wild birds and animals, vegetables from wild plants and mushrooms that were grown on natural logs) from non-marketed sources (**S6 Table**), agreeing with a previous study **[28]**. The ^137^Cs concentration in vegetarian produce and meat/eggs was monitored from 1986–2004, and the concentration reported was below approximately 1 Bq/kg **[29]**. In fact, the concentration of most of the foodstuffs were below 0.2 Bq/kg in 2004 **[13, 29]**. Thus, those foodstuffs exceeding the JML in this study were contaminated by the ^134,137^Cs after the FDNPS accident. The data on all fishery foodstuffs including both those of wild and aquaculture origins examined and reported in the database were further analyzed in detail to find any observable trends in testing and the reported ^134,137^Cs concentrations.

### Analyses of data reported from fishery foodstuffs

**Fig 3:**
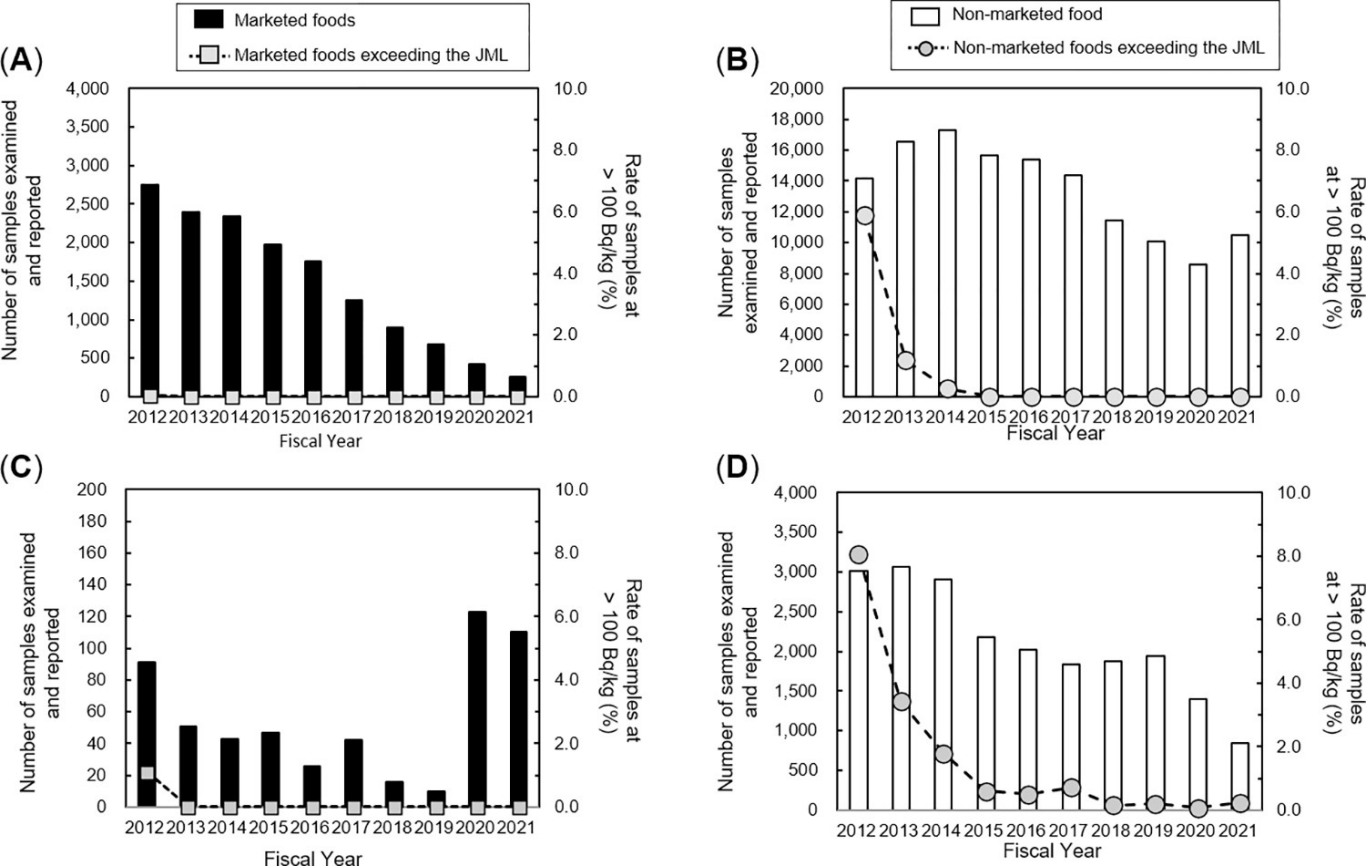
Results on Cs concentration data analyzed for fishery foodstuffs. Annual trends of marketed and non-marketed marine fishery foodstuffs (A and B, respectively) or marketed and non-marketed freshwater fishery foodstuffs (C and D, respectively) reported as exceeding 100 Bq/kg. All data reported using a GE semiconductor detector in the database were included in the analysis.

The data on freshwater fishery foodstuffs and others, including marine fishery foodstuffs, crustaceans and migratory fish, showing a particular change over time were extracted for further analysis according to the scheme shown in **S1 Fig**. The data obtained were then grouped into the categories of marketed/non-marketed and wild/aquaculture. **Fig 3** shows the results from the fishery foodstuffs category grouped into marketed and non-marketed foodstuffs. The rate of non-marketed samples from both freshwater and other fishery foodstuffs reported as exceeding 100 Bq/kg decreased but varied significantly in the FY 2012 to FY 2015 (assuming that the data were collected and tested for ^134,137^Cs concentrations annually under the same conditions throughout Japan; Fisher’s exact test, p > 0.05). From the FY 2012 to FY 2021 reports, one out of 14,723 and 1,074 out of 134,116 marketed and non-marketed marine foodstuffs examined, respectively, were reported to exceed the JML (**S7 Table**). The single case for marketed marine foodstuffs was reported in the FY 2012 (Pacific cod, No. 7272, sampled on 9-8-2012 in Aomori prefecture) with 44 non-marketed marine foodstuffs reported in the FY 2014. In 2015, four cases were reported (White rockfish, Nos. 10159, 15354, 11252, sampled on 6-1-2015, 18-1-2015, 27-1-2015 in Fukushima prefecture; Stone flounder, No. 17317, sampled on 6-3-2015 in Fukushima prefecture) with one recent case in 2021 (Black rockfish, No. 530, sampled on 1-4-2021 in Fukushima prefecture) (**S7 Table**). From the FY 2012 to FY 2021, one out of 559 and 446 out of 21,101 marketed and non-marketed freshwater fishery foodstuffs examined, respectively, were reported to exceed the JML (**S8 Table**). The last marketed example of freshwater fishery foodstuffs (Gin-buna, No. 11914, sampled on 17-4-2012 in Chiba prefecture) was reported in 2012. Fewer than five reports per year (Char, No. 60702, sampled on 12-10-2018 in Fukushima prefecture; Cherry trout, No. 79178, sampled on 1-11-2018 in Fukushima prefecture; Char, No. 4108, sampled on 17-6-201 in Fukushima prefecture; Char, No. 25979, sampled on 7-5-2019 in Gunma prefecture; Cherry trout, No. 25985, sampled on 7-5-2019 in Gunma prefecture; Char, No. 4108, sampled on 17-6-2019 in Fukushima prefecture; Cherry trout, No. 19705, sampled on 8-9-2019 Fukushima prefecture; Char, No. 86, sampled on 12-4-2020 in Gunma prefecture; Char, No. 1918, sampled on 14-10-21 in Fukushima prefecture; Cherry trout, No. 1956, sampled on 27-10-2021 in Fukushima prefecture) were reported to exceed the JML in non-marketed freshwater fishery foodstuffs between 2018 and 2021, although the number of freshwater fishery foodstuffs examined was approximately one tenth of the number of other fishery foodstuffs examined including marine species (**S7 and S8 Tables**). These data indicated that the fishery foodstuffs that were frequently reported as exceeding the JML were non-marketed freshwater fish after FY2015.

### Origin of fishery foodstuffs reported in the database

To examine the origin of fishery foodstuffs contaminated by ^134,137^Cs in more detail, the original information in the database was further filtered into two groups, “aquaculture” or “others” which included “wild”, “not aquaculture”, “blank space [including no data]”, “-”, etc.). The information on freshwater fishery foodstuffs and others including marine fishery foodstuffs was analyzed according to the scheme in **S1 Fig**. **S11 Fig** shows the actual distribution of all the reported fishery foodstuffs examined. There was a notable difference in the proportions exceeding the JML between the aquaculture and wild fishery foodstuffs reported in the database. **Table 2** shows that the number and the proportions of aquaculture fishery foodstuffs for both freshwater and marine fishery foodstuffs that exceeded the JML were lower than those of the wild fishery foodstuffs. Within the aquaculture fishery foodstuffs category, only one sample exceeding the JML was detected (Oriental weather loach, No. 13180, sampled on 18-6-2012 in Fukushima prefecture) from freshwater foodstuffs. A total of 8,231 samples from marine aquaculture fishery foodstuffs were examined, with none exceeding the JML value. From the FY 2015 to FY 2021, only one sample, the wild Black rockfish (*Sebastes schlegelii*) caught off the coast of Fukushima prefecture by an experimental fishing vessel, was reported to have a ^134,137^Cs concentration of 270 Bq/kg (^134^Cs at < 8.9 Bq/kg, ^137^Cs at 270 Bq/kg). In contrast, samples of wild freshwater fishery foodstuffs have been continuously reported in the database as exceeding the JML. However, none of the marketed foodstuffs that exceeded the JML had been distributed on the Japanese market since the FY 2013 (**S7 and S8 Tables**).

**Table 2:** Origin of the examined fishery food products that were reported to exceed 100 Bq/kg*.

Fiscal Year	Aquaculture						Others including wild					
	Freshwater fishery foodstuffs			Others including marine fishery foodstuffs			Freshwater fishery foodstuffs			Others including marine fishery foodstuffs		
	Examined	Exceeded	Rate** (%)	Examined	Exceeded	Rate** (%)	Examined	Exceeded	Rate** (%)	Examined	Exceeded	Rate** (%)
2012	601	1	0.17	210	0	0.00	2,544	243	9.55	18,042	836	4.63
2013	451	0	0.00	456	0	0.00	2,737	105	3.84	19,410	194	1.00
2014	378	0	0.00	1,058	0	0.00	2,630	52	1.98	19,517	44	0.23
2015	302	0	0.00	1013	0	0.00	1,930	13	0.67	17,369	0	0.00
2016	269	0	0.00	910	0	0.00	1,778	10	0.56	16,877	0	0.00
2017	214	0	0.00	974	0	0.00	1,668	13	0.78	15,186	0	0.00
2018	178	0	0.00	874	0	0.00	1,721	3	0.17	11,995	0	0.00
2019	168	0	0.00	807	0	0.00	1,787	4	0.22	10,399	0	0.00
2020	140	0	0.00	834	0	0.00	1,392	1	0.07	8,427	0	0.00
2021	127	0	0.00	1095	0	0.00	832	2	0.24	9,887	1	0.01
Total	2,828	1	0.04	8,231	0	0.00	19,019	446	2.35	147,109	1,075	0.73

*All data using all instruments used including no indicated instrument and a non-destructive method reported in the database were summarized.

**Annual rate exceeding the JML.

**Fig 4:**
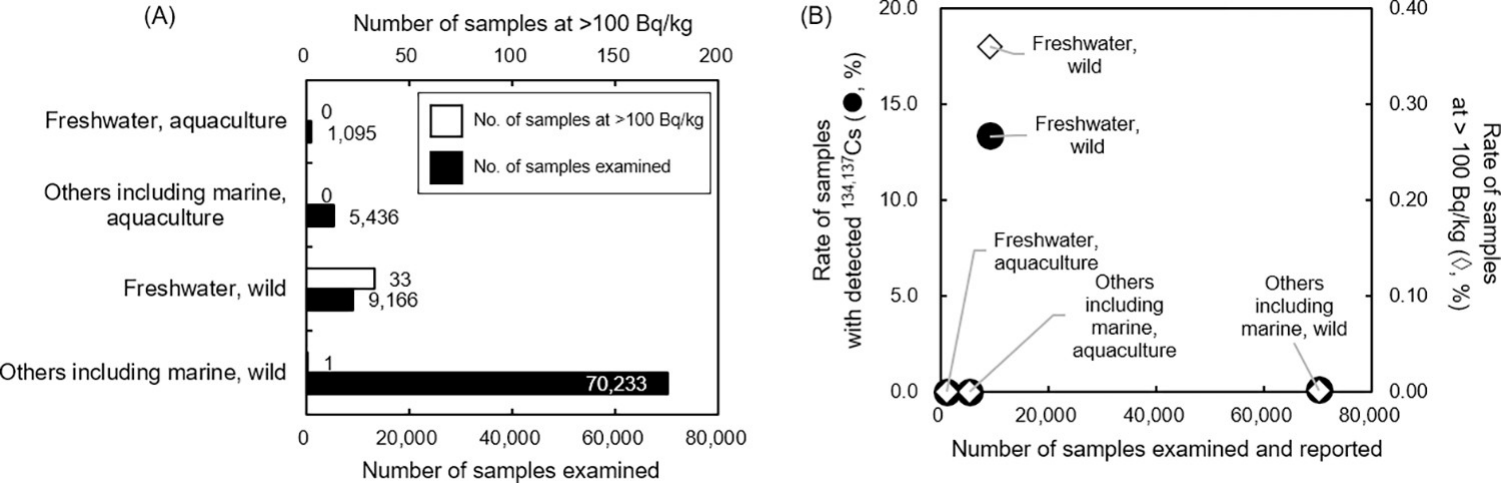
The type of fishery foodstuffs examined and detected ^134,137^Cs during the FY 2016–2020. (**A**) The number of samples examined and number of samples exceeding 100 Bq/kg ^134,137^Cs plotted for wild/aquaculture, marine/freshwater fishery foodstuffs. (**B**) Comparison of the number of fishery foodstuffs examined and the proportions of samples detected with ^134,137^Cs concentration or of samples exceeding the JML.

Those species reported with a ^134,137^Cs concentration above 25 Bq/kg in the database since the FY 2016 are shown in **S9 Table**. These species were consistent with those mentioned in previous reports. The fishery foodstuffs that exceeded the JML were partly concentrated in freshwater areas because of the contamination of the food web in aquatic ecosystems, as reported previously **[4, 30–32]**, and the amount of radiocesium that had accumulated in pelagic fish decreased sharply after the FDNPS accident **[30, 33, 34]**. The outflow and dilution of ^134,137^Cs by ocean currents is thought to be responsible for the undetectable levels of radioactive Cs in marine foodstuffs (bottom layer) in recent years **[35]**, while some sedentary rockfish (*Sebastes cheni*, *Sebastes oblongus*, *Sebastes pachycephalus*, *Sebastes schlegelii* Hilgendorf, 1880) species have been reported with radiation levels of more than 10 kBq/kg-wet in the FDNPS port **[34, 36]**. The original data were grouped according to location (prefecture) (**S10 and S11 Tables**). A total of 21,847 and 155,340 freshwater and other fishery foodstuffs including marine fish foodstuffs, respectively, were examined in the database from FY 2012 to 2021. The number of samples examined has been reported nationwide (27 out of 47 prefectures for freshwater foodstuffs and 42 out of 47 prefectures for other fishery foodstuffs) in Japan since the FY 2012. Testing for ^134,137^Cs in fishery foodstuffs in Japan has focused more closely on marine fishery foodstuffs than on freshwater fishery foodstuffs (**Fig 4, S12 Table**). The fish species (**S9 Table**) and the reported locations tested (**S10 and S11 Tables**) were apparently biased in the ^134,137^Cs testing data. The number of foodstuffs examined, the type of fish tested and where they were tested were not consistently entered within the database. Regarding the examination of the fishery food products, the focus for testing on ^134,137^Cs contamination should be on wild freshwater fish.

Overall, analyzing these data can enable monitoring to be focused more closely on those foodstuff categories where the ^134,137^Cs concentrations are highest and pose the greatest food safety concerns. It would then be possible to reduce the overall number of samples required for determining Cs concentrations by reducing the number of samples from categories known to be of low concern and increase the number from those of high concern. More than a decade after the accident, this would reduce inspection costs, the workload and the waste of precious food sources.

All data reported using a GE semiconductor detector in the database were included in the analysis.

## Conclusions

This comprehensive analysis of a decade of cumulative radiocesium testing data for foodstuffs from the whole of Japan after the 2011 Fukushima Daiichi Nuclear Power Plant accident has for the first time indicated that the levels of ^134,137^Cs have become low in recent years. An exception to this is for foodstuffs that are difficult to cultivate, feed or manage. Japanese foodstuffs show high levels of safety regarding ^134,137^Cs contamination, particularly cultured and aquaculture foodstuffs produced for the market in Japan. Therefore, inspections should be continued with a focus on wild food products that are difficult to cultivate, feed or manage. This approach will provide a successful analysis of the risk of consuming foodstuffs contaminated with radionuclides after an unexpected accident like that at FDNPS.

## Supporting information

S1 Fig Scheme of the analysis of fishery foodstuffs reported in the database. (TIF)

S2 Fig . Scheme of workflow in preparation for data analysis (Steps 1–5). Step 1. Data were obtained from the MHLW homepage (https://www.mhlw.go.jp/stf/kinkyu/0000045250.html, last accessed on July 4, 2022). Step 2. The obtained original monthly data files were integrated to annual data files using Python. Step 3. The obtained original data were reformatted using Python. Step 4. The revised data in.csv files were obtained. Step 5. The obtained.csv files were used for calculation and analysis using R.
(TIF)

S3 Fig Maps of all examined foodstuffs reported during FY 2012–2021. Prefectural locations are indicated by border-lines. Prefectures having no foods reported were left clear. The location of FDNPS is indicated by an orange dot. All data reported in the database were included in the analysis.
(TIF)

S4 Fig Maps of all examined “general foodstuffs” reported during FY 2012–2021. Prefectural locations are indicated by border-lines. Prefectures having no foods reported were left clear. The location of FDNPS is indicated by an orange dot. All data reported in the database were included in the analysis.
(TIF)

S5 Fig Maps of all examined “milk and infant foodstuffs” reported during FY 2012–2021. Prefectural locations are indicated by border-lines. Prefectures having no foods reported were left clear. The location of FDNPS is indicated by an orange dot. All data reported in the database were included in the analysis.
(TIF)

S6 Fig Maps of all examined “drinking water including soft drinks containing tea as a raw material” reported during FY 2012–2021. Prefectural locations are indicated by border-lines. Prefectures having no foods reported were left clear. The location of FDNPS is indicated by an orange dot. All data reported in the database were included in the analysis.
(TIF)

S7 Fig Summary of ^134,137^Cs concentration reported for foodstuffs in the “general foodstuffs” category. (A) Distribution curve of ^134,137^Cs concentration. Samples exceeding the JML (10 Bq/kg, 50 Bq/kg and 100 Bq/kg for “drinking water including soft drinks containing tea as a raw material,” “milk and infant foodstuffs,” and “general foodstuffs,” respectively) are in red, while the others are in grey. (B) Violin plot of ^134,137^Cs concentration (without the inequality sign) in each year. The table indicates the concentration of samples exceeding or within the JML. All data reported in the database were included in the analysis.
(TIF)

S8 Fig The reported instruments used to detect Cs concentration in foodstuffs. Ge, germanium semiconductor detector; CsI&NaI, gamma spectrometry with sodium iodide, NaI(Tl), and cesium iodine, CsI(Tl), scintillation detectors; -, including no indicated information and a non-destructive method. Data from the meat testing from all livestock were excluded from analysis.
(TIF)

S9 Fig Analysis of marketed and non-marketed foodstuffs during FY 2012–2021. The number and ratio of all marketed and non-marketed food products in each year were plotted in the graph. Data from cattle meats from all livestock testing practice were excluded from analysis.
(TIF)

S10 Fig Rate of foodstuffs exceeding the JML in the “general foodstuffs” category. The pie graph shows the rate of foodstuffs reported at > 100 Bq/kg in the “general foodstuffs" category during the FY 2012–2021. The number and rate of the “general foodstuffs” reported are summarized in the table. All data reported in the database were included in the analysis.
(TIF)

S11 Fig Results of Cs concentration data analyzed for the reported fishery foodstuffs. Annual trends of aquaculture or wild marine fishery foodstuffs (A or B, respectively) and aquaculture or wild freshwater fishery foodstuffs (C and D, respectively) examined and reported exceeding the JML (100 Bq/kg). All data reported in the database were included in the analysis.
(TIF)

S1 Table Number of foodstuffs in all categories reported by each prefecture in Japan. (XLSX)

S2 Table Number of “general foodstuffs” reported by each prefecture in Japan. (XLSX)

S3 Table Number of the “milk and infant foodstuffs” reported by each prefecture in Japan. (XLSX)

S4 Table Number of “drinking water including soft drinks containing tea as a raw material” reported by each prefecture in Japan. (XLSX)

S5 Table Summary of ^134,137^Cs concentration in foodstuffs reported during the FY 2012–2021*. (XLSX)

S6 Table List of the foodstuffs in the “general foodstuffs” category reported to exceed the JML (100 Bq/kg). (XLSX)

S7 Table Results of ^134,137^Cs monitoring for the fishery foodstuffs excluding freshwater foodstuffs*. (XLSX)

S8 Table Results of ^134,137^Cs monitoring for the freshwater fishery foodstuffs*. (XLSX)

S9 Table List of fishery foodstuffs reported to have a ^134,137^Cs concentration >25 Bq/kg during the FY 2016–2021*. (XLSX)

S10 Table Data of freshwater fishery foodstuffs reported and sorted according to the prefectural government in Japan*. (XLSX)

S11 Table Data of the other fishery foodstuffs including marine fishery foodstuffs reported and sorted according to the prefectural government in Japan*. (XLSX)

S12 Table Reported ^134,137^Cs concentration in fishery foodstuffs during the FY 2016–2021*. (XLSX)
