# Supplementary figures and images for: Comprehensive analysis of a decade of cumulative radiocesium testing data for foodstuffs throughout Japan after the 2011 Fukushima Daiichi Nuclear Power Plant accident
Source: PLoS One. 2022 Sep 21;17(9):e0274070. doi: 10.1371/journal.pone.0274070 (PMC9491560; doi:10.1371/journal.pone.0274070)

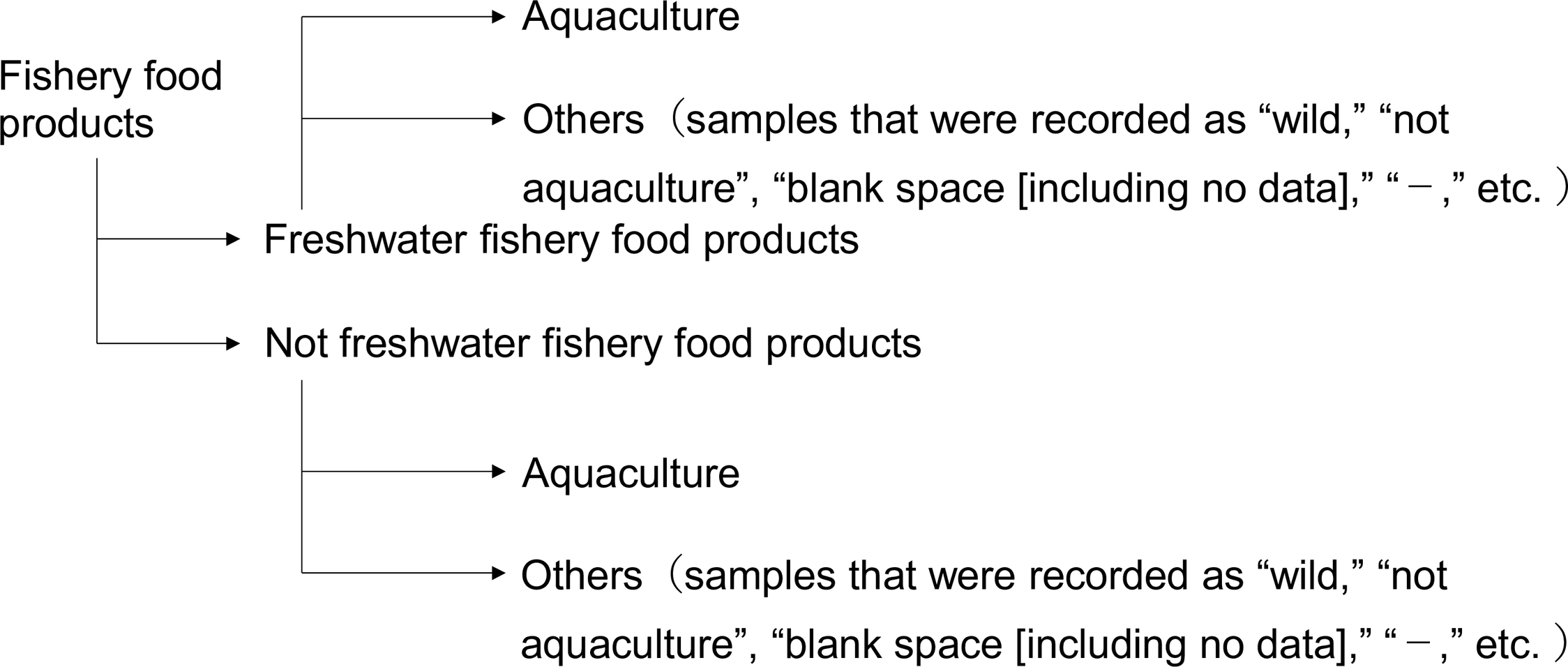

Supplement: S1 Fig — (TIF) [file pone.0274070.s001.tif]

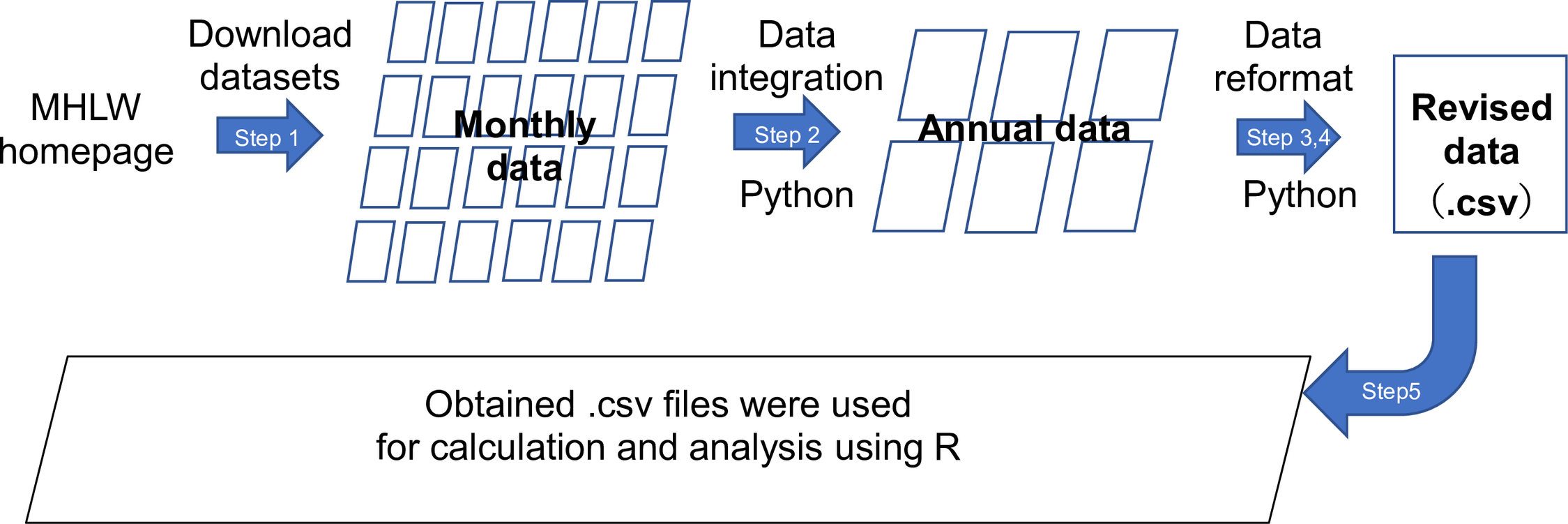

Supplement: S2 Fig — Step 1. Data were obtained from the MHLW homepage (https://www.mhlw.go.jp/stf/kinkyu/0000045250.html, last accessed on July 4, 2022). Step 2. The obtained original monthly data files were integrated to annual data files using Python. Step 3. The obtained original data were reformatted using Python. Step 4. The revised data in.csv files were obtained. Step 5. The obtained.csv files were used for calculation and analysis using R. (TIF) [file pone.0274070.s002.tif]

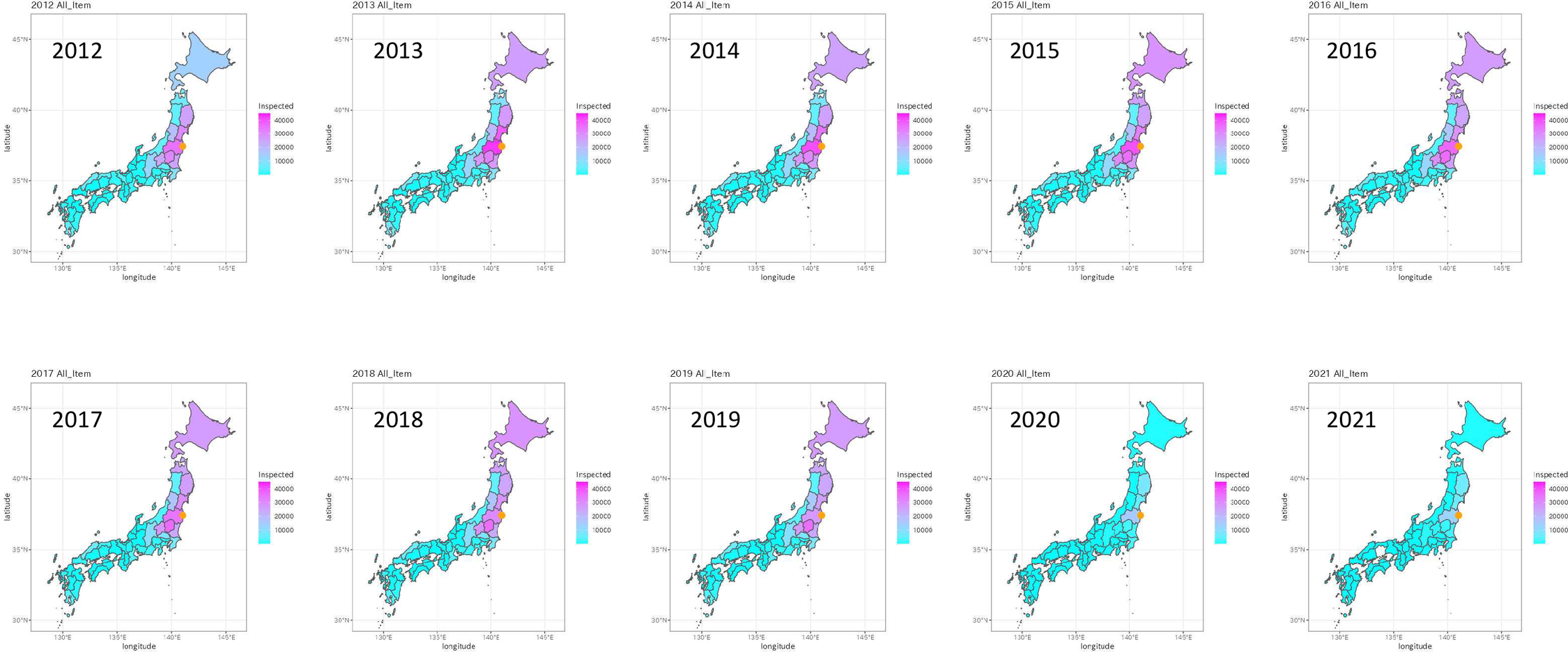

Supplement: S3 Fig — Prefectural locations are indicated by border-lines. Prefectures having no foods reported were left clear. The location of FDNPS is indicated by an orange dot. All data reported in the database were included in the analysis. (TIF) [file pone.0274070.s003.tif]

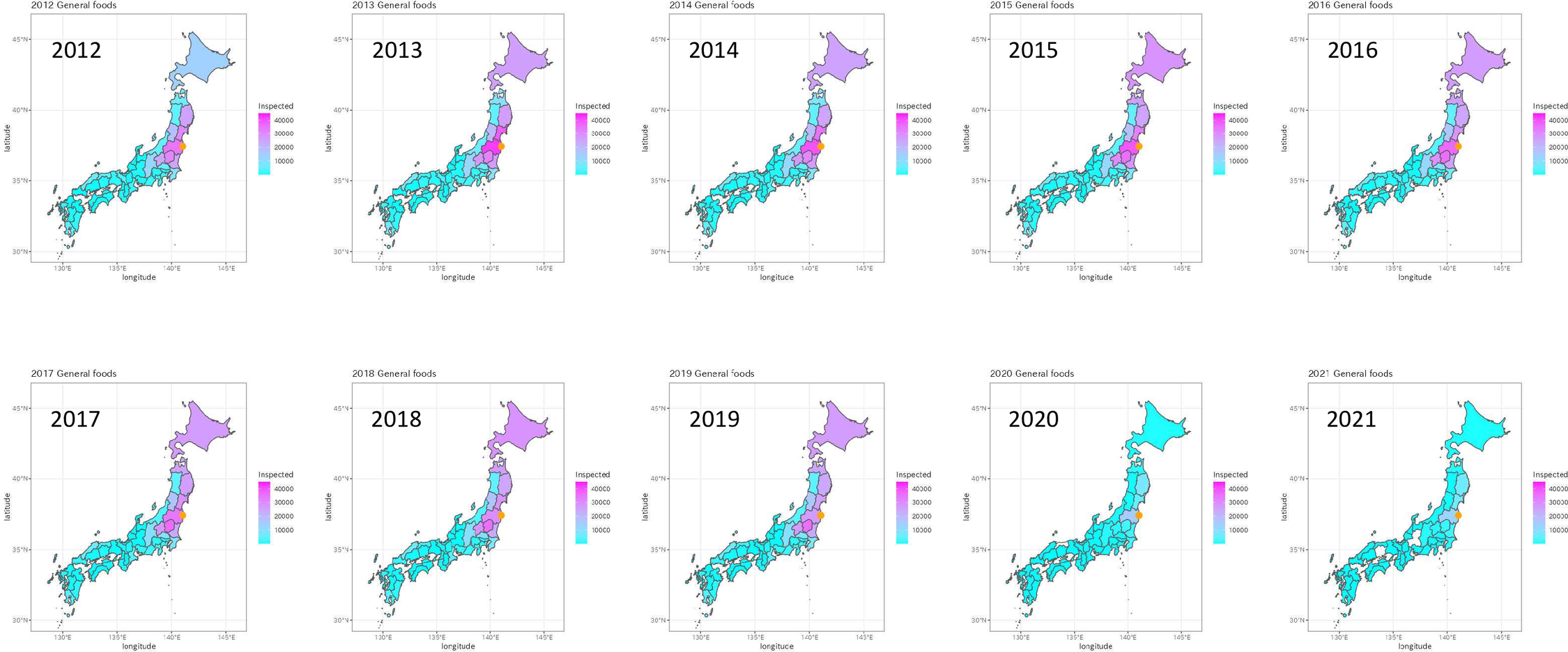

Supplement: S4 Fig — Prefectural locations are indicated by border-lines. Prefectures having no foods reported were left clear. The location of FDNPS is indicated by an orange dot. All data reported in the database were included in the analysis. (TIF) [file pone.0274070.s004.tif]

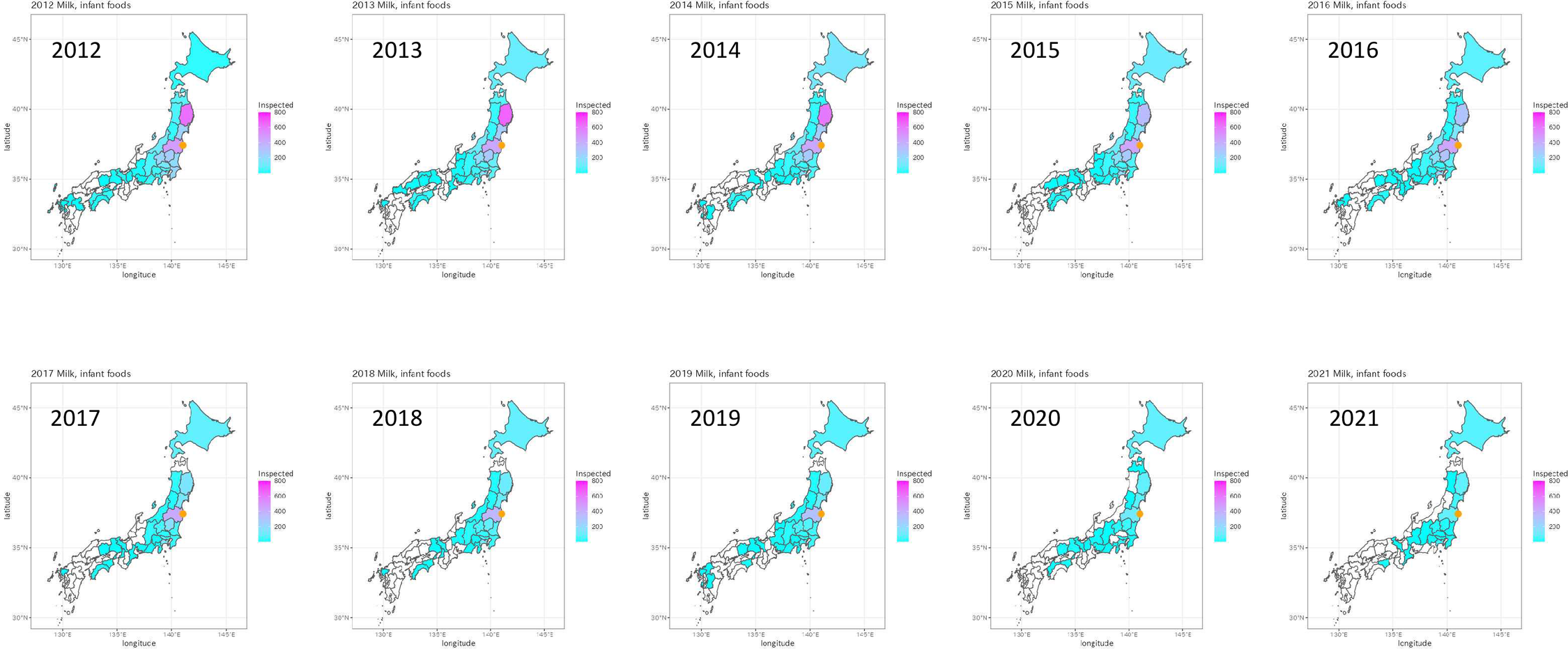

Supplement: S5 Fig — Prefectural locations are indicated by border-lines. Prefectures having no foods reported were left clear. The location of FDNPS is indicated by an orange dot. All data reported in the database were included in the analysis. (TIF) [file pone.0274070.s005.tif]

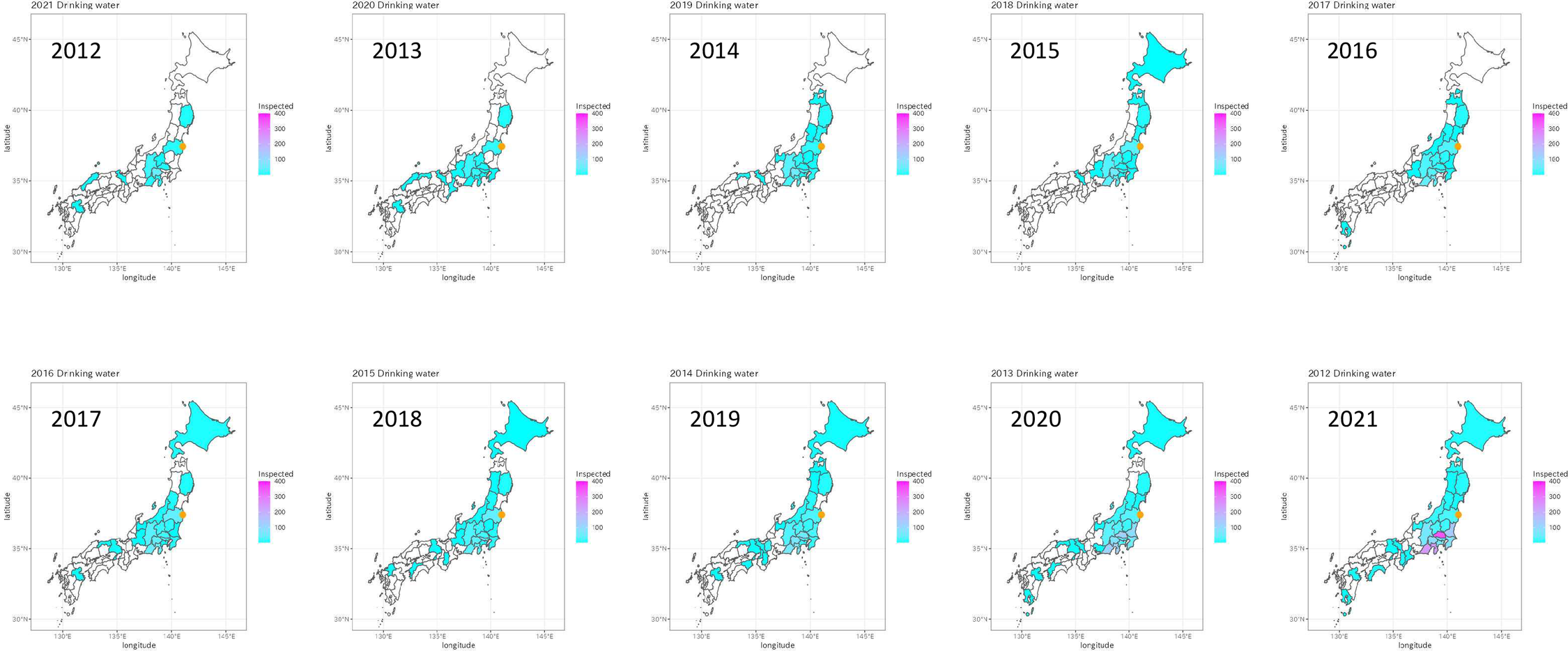

Supplement: S6 Fig — Prefectural locations are indicated by border-lines. Prefectures having no foods reported were left clear. The location of FDNPS is indicated by an orange dot. All data reported in the database were included in the analysis. (TIF) [file pone.0274070.s006.tif]

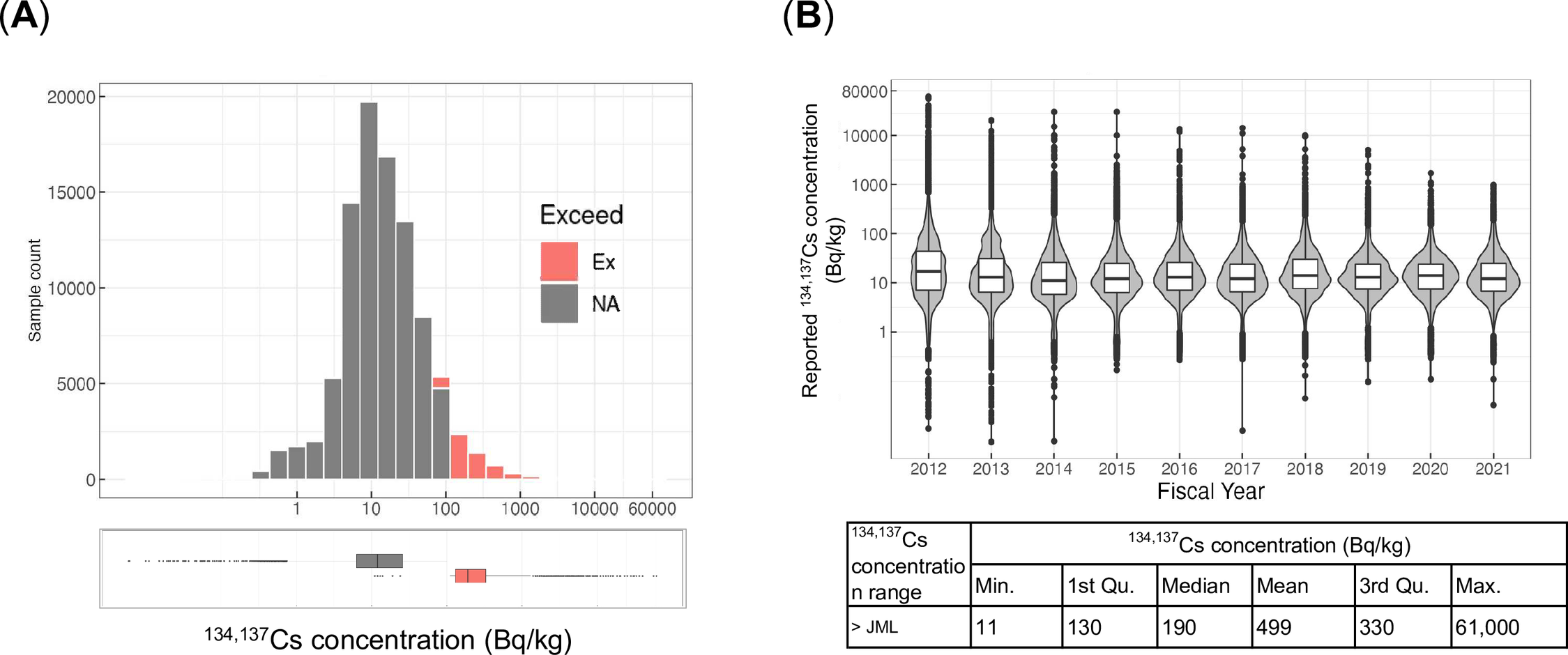

Supplement: S7 Fig — (A) Distribution curve of 134,137Cs concentration. Samples exceeding the JML (10 Bq/kg, 50 Bq/kg and 100 Bq/kg for “drinking water including soft drinks containing tea as a raw material,” “milk and infant foodstuffs,” and “general foodstuffs,” respectively) are in red, while the others are in grey. (B) Violin plot of 134,137Cs concentration (without the inequality sign) in each year. The table indicates the concentration of samples exceeding or within the JML. All data reported in the database were included in the analysis. (TIF) [file pone.0274070.s007.tif]

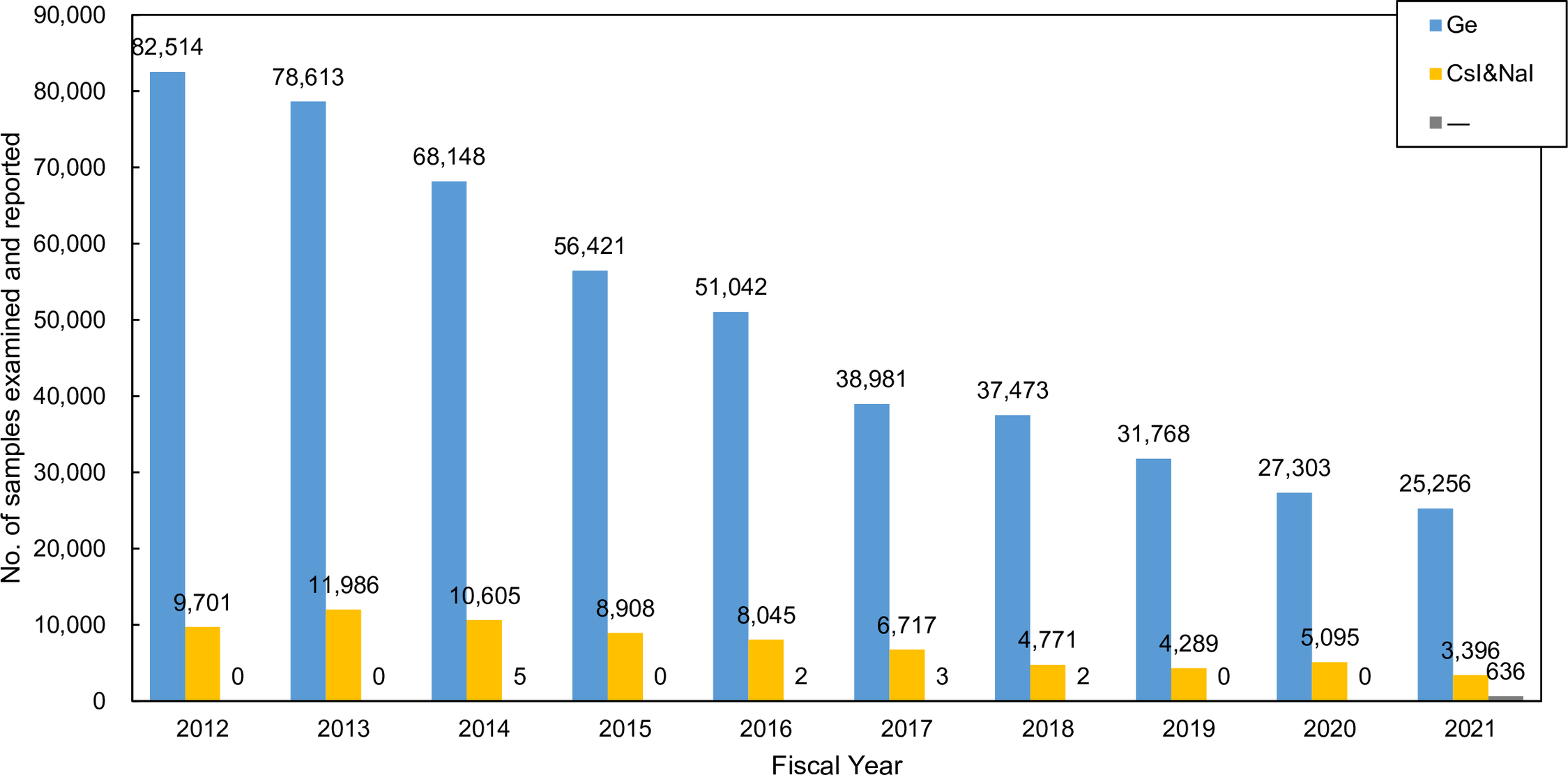

Supplement: S8 Fig — Ge, germanium semiconductor detector; CsI&NaI, gamma spectrometry with sodium iodide, NaI(Tl), and cesium iodine, CsI(Tl), scintillation detectors; -, including no indicated information and a non-destructive method. Data from the meat testing from all livestock were excluded from analysis. (TIF) [file pone.0274070.s008.tif]

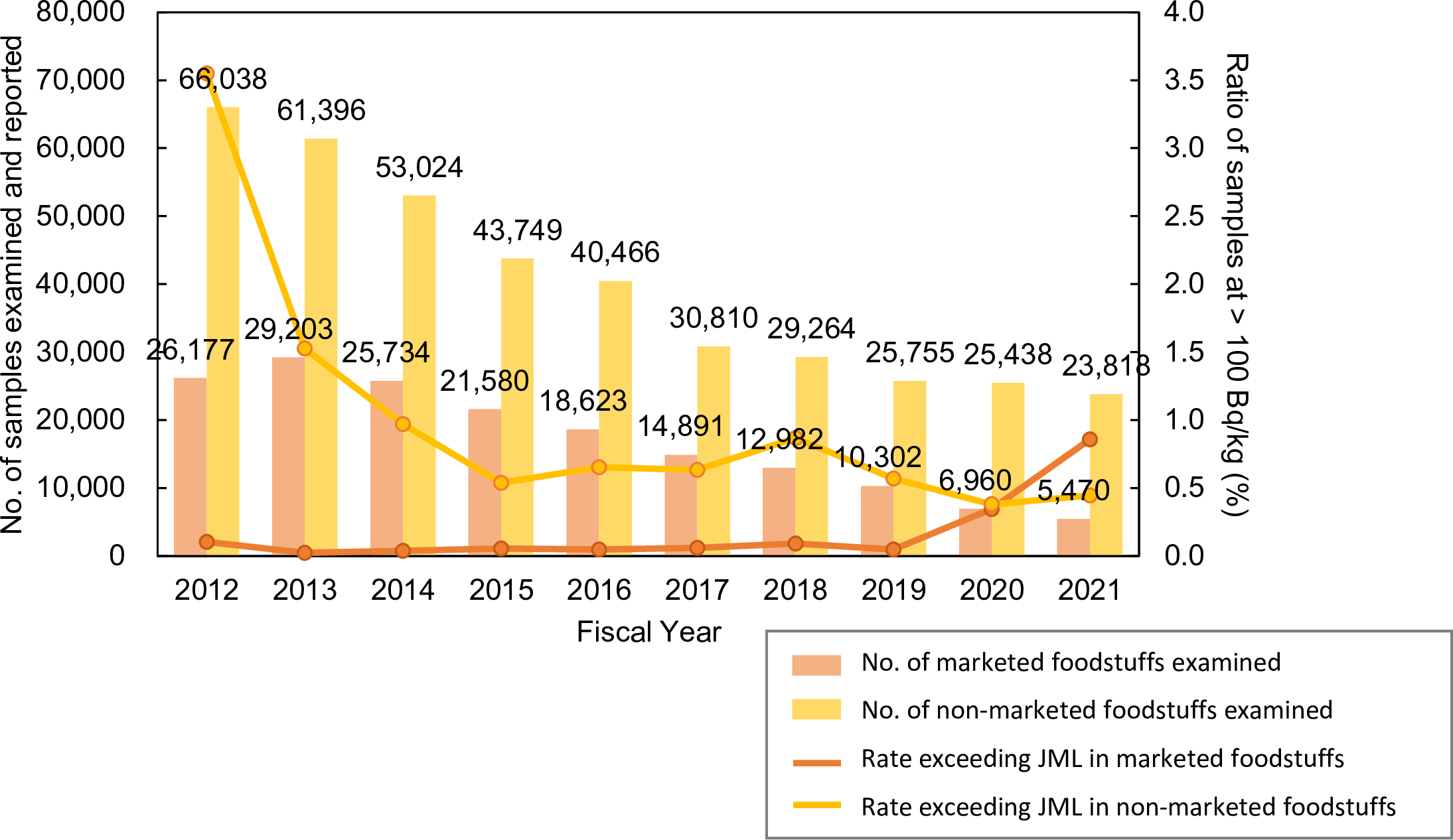

Supplement: S9 Fig — The number and ratio of all marketed and non-marketed food products in each year were plotted in the graph. Data from cattle meats from all livestock testing practice were excluded from analysis. (TIF) [file pone.0274070.s009.tif]

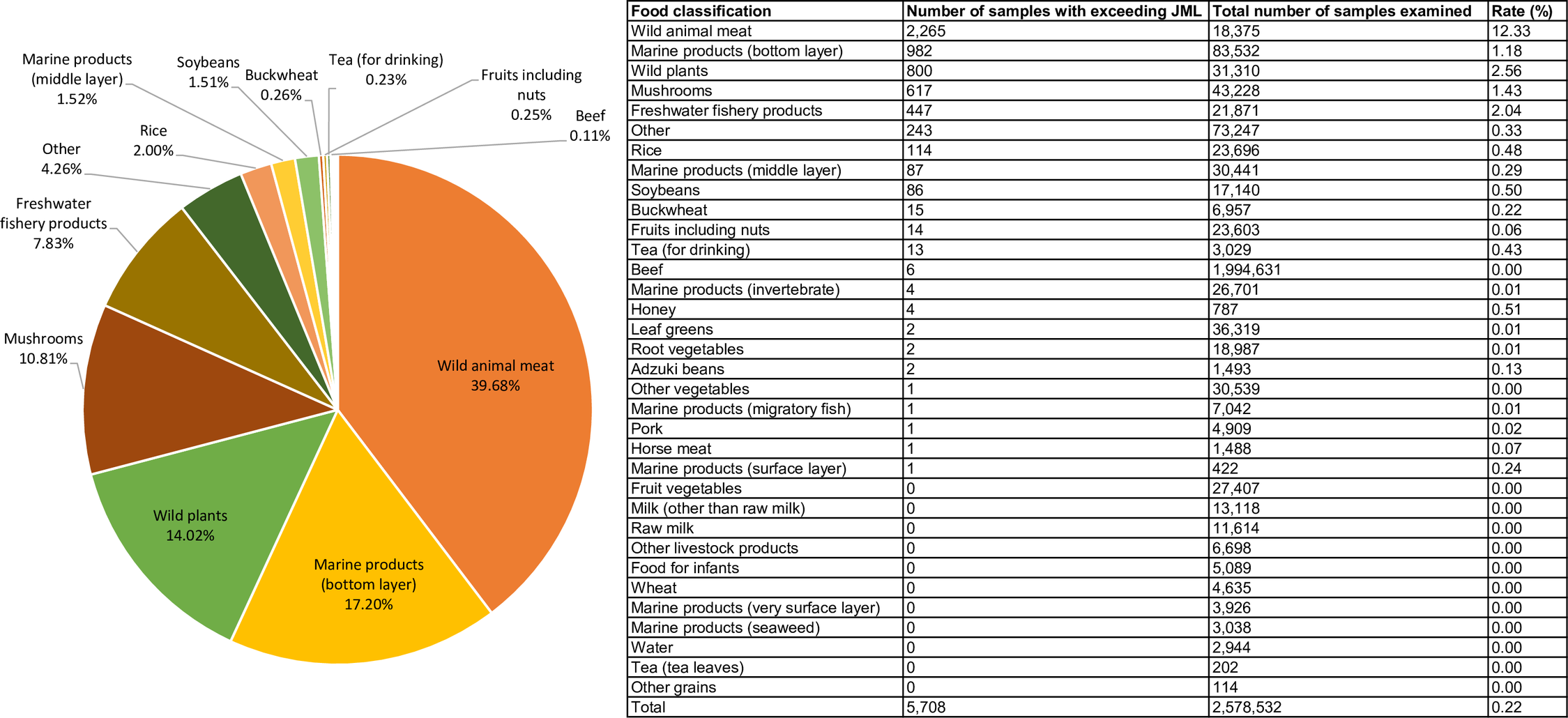

Supplement: S10 Fig — The pie graph shows the rate of foodstuffs reported at > 100 Bq/kg in the “general foodstuffs" category during the FY 2012–2021. The number and rate of the “general foodstuffs” reported are summarized in the table. All data reported in the database were included in the analysis. (TIF) [file pone.0274070.s010.tif]

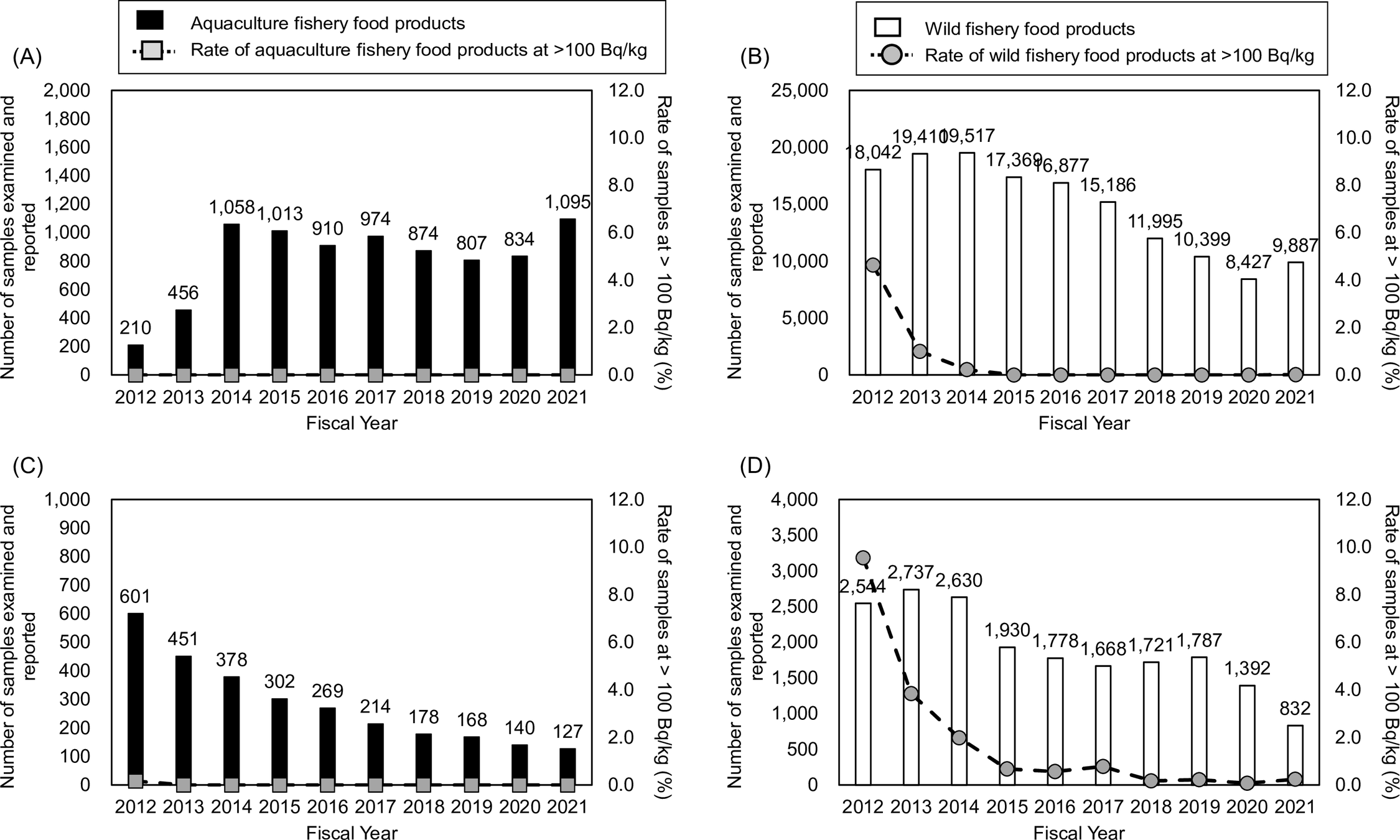

Supplement: S11 Fig — Annual trends of aquaculture or wild marine fishery foodstuffs (A or B, respectively) and aquaculture or wild freshwater fishery foodstuffs (C and D, respectively) examined and reported exceeding the JML (100 Bq/kg). All data reported in the database were included in the analysis. (TIF) [file pone.0274070.s011.tif]
